# Assessment of the Antitumor Activity of Green Biosynthesized Zinc Nanoparticles as Therapeutic Agent Against Renal Cancer in Rats

**DOI:** 10.1007/s12011-022-03126-5

**Published:** 2022-01-26

**Authors:** Sawsan El-Sonbaty, Eman I. Kandil, Riham Abdel-Hamid Haroun

**Affiliations:** 1 Department of Radiation Microbiology, National Center for Radiation Research and Technology, Atomic Energy Authority, Cairo, Egypt; 2 Faculty of Science, Department of Biochemistry, Ain Shams University, Cairo, Egypt

**Keywords:** Renal cell carcinoma, Ferric-nitrilotriacetate (Fe-NTA), Nanoparticles, Green biosynthesis, Zinc nanoparticles (Zn-NPs), White mushroom

## Abstract

Zinc nanoparticles (Zn-NPs) have garnered a great deal of attention as potential cancer therapy. The use of microorganisms in the synthesis of nanoparticles emerges as an eco-friendly and exciting approach. This study was designed to assess biosynthesized Zn-NPs as therapeutic agent against kidney cancer induced by ferric-nitrilotriacetate (Fe-NTA) in rats.

Zn-NPs were synthesized from edible mushroom then characterized by transmission electron microscopy analysis, dynamic light scattering, and Fourier transform infrared spectroscopy. Rats were divided into 4 different groups: group I (control), group II (Fe-NTA group), group III (Zn-NPs group), and group IV (Fe-NTA + Zn-NPs group). Animals were sacrificed then kidney and liver function tests, MDA level, glutathione, glutathione peroxidase, and superoxide dismutase activities were measured by using colorimetric methods. Caspase-3 level and carcinoembryonic antigen concentration were measured by using ELISA. Finally, DNA fragmentation was visualized by using agarose gel electrophoresis.

Treatment with Zn-NPs significantly suppressed renal oxidative stress by restoring glutathione level, glutathione peroxidase, and superoxide dismutase activities and ameliorated oxidative damage parameters of lipid peroxidation as well as renal toxicity markers. Molecular and tumor markers showed significant improvement with respect to induction group, and this was well appreciated with the histopathological alteration findings in the treated groups.

Microbial synthesized Zn-NPs possess antitumor-promoting activity against Fe-NTA-induced toxicity and carcinogenesis, which should be evaluated in a clinical study.

## Introduction

Cancer is reported as the second, after cardiovascular diseases, leading cause of death. In 2018, there were about 18.1 million new cancer cases and 9.6 million cancer deaths worldwide [1]. Renal cell carcinoma (RCC) is recorded as the third most frequent urologic malignancy, affecting more than 400,000 patients each year [2]. Due to the increasing prevalence of malignancies, new effective therapeutics and treatment strategies are highly required [1]. Nowadays, there is a growing interest in applying the nanotechnology to cancer due to its uniquely appealing features for diagnosis and imaging, drug delivery, and the therapeutic nature of some nanomaterials themselves [3]. There are different chemical and physical methods for the nanoparticle production, which are still being under investigation for the purpose of obtaining particles with a certain size and lower toxicity [4]. Green synthesis of nanoparticles, in which the nanoparticles can be biologically synthesized by using living organisms such as bacteria, fungi, and plants, is considered a new approach to prevent the production of unsafe or undesired byproducts by reliable, maintainable, and eco-favorable synthesis techniques. [5, 6].

Zinc is an essential trace element that is crucial for growth and development; it has three major biological functions, as catalyst, structural, and regulatory functions as it is representing an integral component of approximately 10% of the human proteome and encompassing hundreds of key enzymes and transcription factors. Moreover, zinc has critical effect in immune function, oxidative stress, and apoptosis [7, 8]. Zinc oxide nanoparticles (ZnO-NPs) are widely used in biomedicine, especially as anticancer and antibacterial, due to their potent ability to induce excess reactive oxygen species (ROS) production, release zinc ions, and induce cell apoptosis [9]. Several studies demonstrated the antitumor activity of ZnO-NPs [10–12], which also proved to have cytotoxic and genotoxic effects [13–16]. To our knowledge, until now, there are no studies designed to investigate the antitumor efficacy of zinc nanoparticles (Zn-NPs), which may be safer than ZnO-NPs. Therefore, the current study was aimed to assess the activity of green biosynthesized Zn-NPs as therapeutic agent against renal cancer induced by ferric-nitrilotriacetate (Fe-NTA) in rats by studying its various potential biochemical and molecular targets as well as tumor promotion markers which are known to be dysregulated in cancer cells.

## Materials and Methods

### Ethics Statement

The current study was approved by the animal ethics care committee of the National Centre for Radiation Research and Technology (NCRRT), Egyptian Atomic Energy Authority, Cairo, Egypt.

### Chemicals

Various chemicals were used in this study, such as disodium salt of nitrilotriacetic acid (NTA), ferric nitrate hydrate, sodium bicarbonate, zinc chloride (ZnCl_2_), and agarose, were procured from Sigma Pvt. Ltd.

### Animals

Adult male Wister rats weighing about 140–170 g purchased from the breeding unit of the Egyptian Organization for Biological Products and Vaccines (Cairo, Egypt) were used in this study. The animals were acclimatized to the laboratory conditions prior to the study for seven days and kept at 25 ± 2 °C and a relative humidity of 40–45% with alternative day and night cycles of 12 h each. They were fed with normal pelleted rat chow and water ad libitum. The normal pelleted rat chow purchased from Al Qaedِِ Company, Cairo, Egypt, contains 65% carbohydrates (corn starch 15% and sucrose 50%), 20.3% proteins (casein 20% and DL-methionine 0.3%), 5% fat (corn oil 5%), 5% fibers, 3.7% salt mixture, and 1% vitamins mixture. Animal maintenance and treatments were conducted in accordance with the National Institute of Health Guide for Animal, as approved by Institutional Animal Care and Use Committee (IACUC). A 30-day toxicity study of Zn-NPs was conducted in male rats by studying plasma biological markers, survival, and decrease in body weights.

### Methods

#### Chemical Studies

##### Preparation of Fe-NTA Solution

Ferric-nitrilotriacetate (Fe-NTA) solution was prepared according to Athar and Iqbal [17]. In brief, disodium salt of nitrilotriacetic acid (NTA) (0.64 mmol/kg body weight (b.w.)) and ferric nitrate hydrate (0.16 mmol/kg b.w.) were dissolved in distilled water, and pH was adjusted to 7.0 using sodium bicarbonate. The molar ratio of Fe to NTA was 1:4.

##### Biosynthesis of Zinc Nanoparticles

Zn-NPs were synthesized according to the method of Philip [18]. Briefly, the edible white mushroom *Agaricus bisporus* was obtained from Ploshia Mushroom Company (Egypt) and washed several times with deionized water; then, about 68 g of finely cut mushroom was boiled for 2 min in 300 ml water and filtered and cooled to room temperature and used as reducing agent and stabilizer. To a vigorously stirred 30 ml aqueous solution of ZnCl_2_ (50 mg/100 ml water), 6 ml mushroom extract was added, and the stirring continued in a shaker at 37 °C and 200 rpm for 24 h for the synthesis of nanoparticles. Finally, the resulting solution was filtered through a 0.22-μm filter (Millipore).

##### Characterization of Zn-NPs

1. Transmission electron microscopy analysis (TEM)

Synthesized Zn-NPs were analyzed by TEM. Samples of Zn-NPs were prepared by placing a drop of the suspension of Zn-NPs on carbon-coated copper grids and allowing water to evaporate. The shape and size of nanoparticles were determined from TEM micrographs. The software (Advanced Microscopy Techniques, Danvers, USA) for the digital TEM camera was calibrated for size measurements of the nanoparticles. TEM measurements were performed on a JEOL model 1200EX.

2. Dynamic light scattering (DLS) of Zn-NPs

Sample of Zn-NPs was analyzed for size determination by DLS Zetasizer (ZS) which was manufactured in Malvern, UK.

3. Fourier transform infrared spectroscopy (FTIR)

Samples of Zn-NPs and biological material used in the synthesis (mushroom extract) were analyzed for functional groups by FTIR, VERTEX-70, BRUKER, Ettlingen, Germany.

4. Determination of LD50 of Zn-NPs using experimental animals

In screening as a new drug, determination of LD_50_ of Zn-NPs is usually an initial step in the assessment and evaluation of its toxic characteristics in vivo and provides information on health hazards likely to arise from short-term exposure to ZnNPs. LD50 of ZnNPs was determined as described by Akhila et al. [19]. According to our pilot study, to estimate LD_50_ of Zn-NPs, various doses of the green biosynthesized Zn-NPs prepared in distilled water were administered once daily by oral gavage. The animals were observed for 30 days, and cumulative mortality within these days was used for the calculation of LD_50_ as the measurement of acute toxicity.

##### Evaluation of Therapeutic Antitumor Efficacy of Biosynthesized Zn-NPs

The present study was designed to comprise a series of in vitro and in vivo investigations, as follows:

1. In vitro study

The antitumor effect and inhibitory concentration 50 (${\mathrm{IC}}_{50}$) of Zn-NPs was investigated on the viability of baby hamster kidney fibroblast cell line (BHK-21) using sulforhodamine-B (SRB) assay for cytotoxicity screening according to the method of Vichai and Kirtikara [20].

2. In vivo study

The long-term in vivo study shows the therapeutic antitumor efficacy of Zn-NPs against chemically induced renal carcinogenesis in the animal model represented in biochemical changes such as changes in antioxidant enzyme activities, lipid peroxidation levels, tumor marker, caspase-3 activity, liver, and kidney functions, as well as histopathological changes following 1-month treatment.

- Experimental design

Sixty animals were randomly divided into four equal groups as follows: in group I (control group), animals in this group were not given any chemical treatment. In group II (Fe-NTA group), rats in this group were intraperitoneally injected with Fe-NTA twice weekly at a dose (9 mg/kg b.w.) until the end of experiment (9 months), and within the period of experiment, diethylnitrosamine will also be injected (200 mg/kg b.w.) once a time. In group III (Zn-NPs group), rats in this group were treated with Zn-NPs orally three times a week, day, or other days at a dose level of 100 µg/kg b.w. for 2 months. In group IV (Fe-NTA + Zn-NPs group), rats in this group were received Fe-NTA as mentioned in group II then treated with Zn-NPs as described in group III. Finally, all the animals were sacrificed, and blood samples were drawn from the vena cava into heparinized syringe, and plasma was separated by centrifugation at 3000* g* for 10 min at 4 °C and stored at − 20 °C pending analyses. Kidneys were excised, washed in ice-cold saline, and blotted to dryness. Each rat kidney was divided into two parts: one put in 10% formalin for histopathological examination and the other one was homogenized in ice-cold 50 mM Tris–HCl/0.25 M sucrose buffer (pH 7.4) to prepare 10% (w/v) tissue homogenate and stored at − 80 °C for analysis of DNA fragmentation and biochemical assays. An aliquot of the kidney tissue homogenate was centrifuged at 10,000 rpm for 15 min at 4 °C, and the cytosolic supernatant was used for the determination of apoptotic agent caspase-3.

b. Biochemical analysis

- Kidney and liver function tests

Plasma creatinine, urea, alanine aminotransferase (ALT), gamma-glutamyl transferase (GGT), lactate dehydrogenase (LDH), sodium (Na +), potassium (K +), albumin, and total proteins were measured colorimetrically using a commercial assay kit (Biodiagnostic, Egypt).

- Determination of malondialdehyde (MDA)
  Lipid peroxide concentrations were determined in kidney tissue homogenate MDA which is the end product of unsaturated fatty acid peroxidation and can react with thiobarbituric acid (TBA) to form a colored complex called thiobarbituric acid-reactive substance (TBARS). TBA reactivity was assayed by the method of Yoshioka et al. [21].
- Glutathione (GSH), glutathione peroxidase (Gpx), and superoxide dismutase (SOD) activities
  Levels of GSH and Gpx and SOD activities were assayed also in kidney tissues by using colorimetric assay kit of Biotech Inc.
- Caspase-3 level
  Homogenates of kidney tissues of each group were assayed for caspase-3 level using caspase-3 colorimetric detection ELISA kit purchased from KOMA Biotech Inc. (South Korea).
- Carcinoembryonic antigen (CEA) concentration
  CEA concentration was determined in plasma using ELISA kit (MBS720630, My Biosource, San Diego, CA, USA).
- DNA fragmentation assay

DNA was extracted from kidney samples as previously described by Bortner et al. [22]. The extent of DNA fragmentation was assessed by the agarose gel electrophoresis containing ethidium bromide (0.5 mg/ml) at 5 V/cm for 1 h.

Histopathological examination

Kidney tissues were fixed in 10% formalin, dehydrated by ethanol, and embedded in paraffin. Sections of 5-μm thickness were cut and stained with hematoxylin and eosin according to the method described by Banchroft et al. [23] and examined by light microscope.

### Statistical Analysis

Statistical analysis was performed using IBM SPSS software (version 23.0; IBM Corp., Armonk, NY, USA). Data are reported as the mean ± SE. One-way ANOVA was used to determine statistically significant differences between group’s means. The level of significance between mean values was set at *P* ≤ 0.05.

## Results

### Characterization of Zn-NPs

#### By TEM

**Fig. 1:**
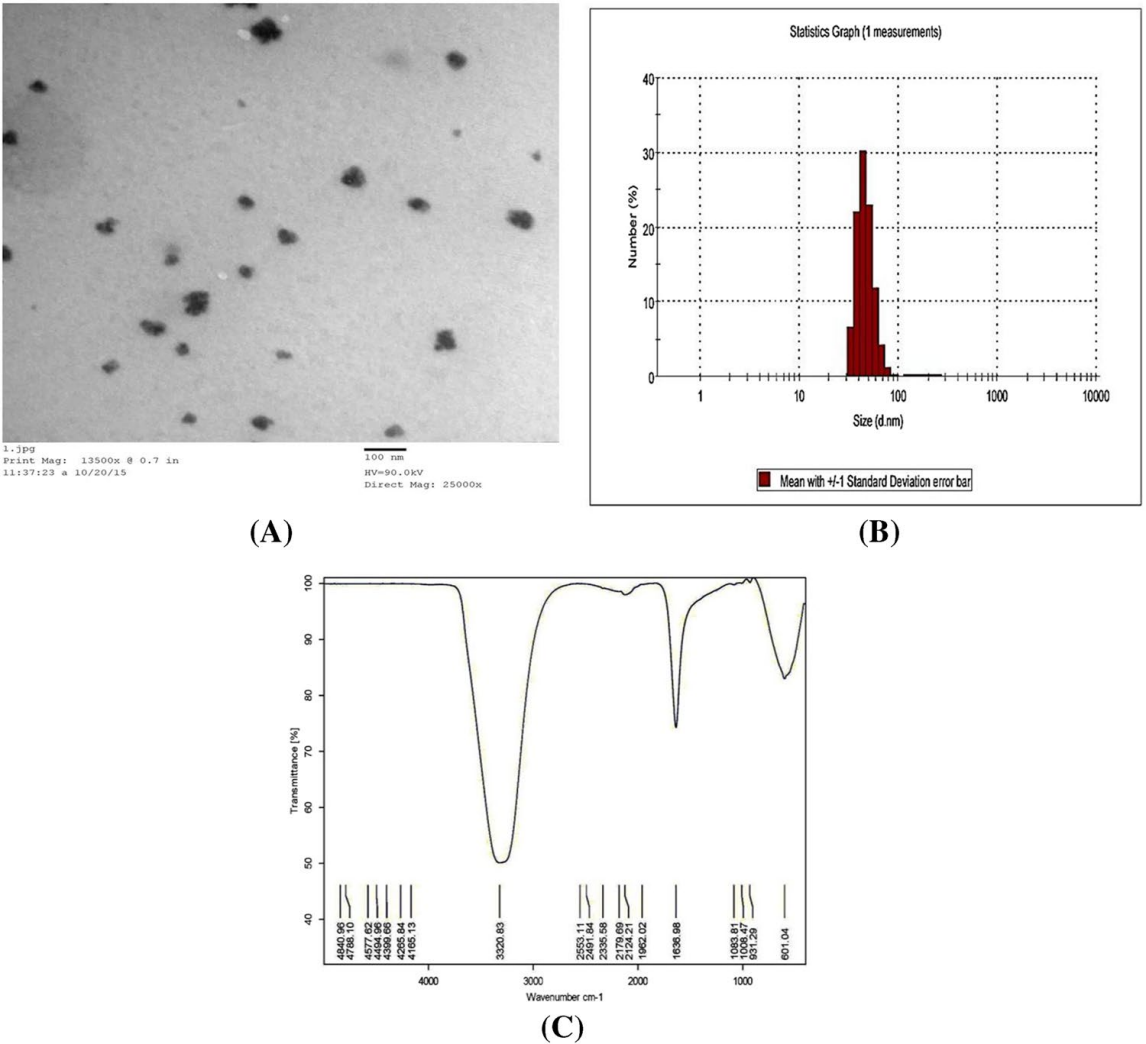
Characterization of Zn-NPs. **A** Transmission electron microscope (TEM) of Zn-NPs. **B** DLS analysis of Zn-NPs for size determination. **C** FTIR Spectroscopy of Zn-NPs

As shown in Fig. 1A, the results of TEM for Zn-NPs showed that Zn-NPs were of different size about (12–17 nm) and different shapes mostly spherical.

#### By DLS

Dynamic light scattering was used for Zn-NPs size determination, which revealed that Zn-NPs size ranged from 50 to 100 nm; 22% of Zn-NPs represented the size of 50 nm, 24% represented 70 nm, and 30% represented 60 nm, as shown in Fig. 1B.

#### By FTIR

FTIR spectrum was used to analyze the biological extract of mushroom used in the synthesis of Zn-NPs and the synthesized Zn-NPs. FTIR analysis of the mushroom extract shows a strong signal at 3320.83 cm^−1^ which represent hydroxyl group (–OH), and at 1636.98 cm^−1^ represents amid I group of proteins and other signals at 601.04 represents single bond of carbon with –H or –N which with other weak bonds named fingerprint (Fig. 1C). FTIR spectrum of Zn-NPs revealed a strong signal at 3324.57 represents –OH, and at 1636.34 represents amid I of proteins, and at 603.38 represents carbon with –H or –N which with other weak bonds named fingerprint (Fig. 3b).

#### Determination of LD_50_ of Zn-NPs

Rats were observed for toxicity and mortality. The result showed that Zn-NPs administration caused no toxicity symptoms, and no mortality was recorded; even Zn-NPs were used at a dose of 1000 µg/kg b.w., and finally, Zn-NPs were used at a dose of 100 µg/kg b.w.

### Evaluation of Therapeutic Antitumor Efficacy of Biosynthesized Zn-NPs

#### In Vitro Results (Cell Viability Tests)

**Fig. 2:**
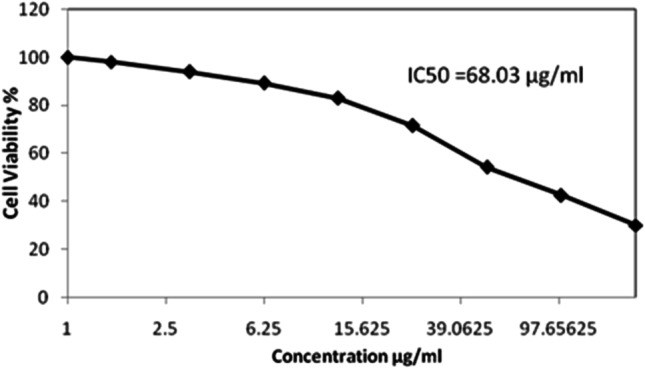
Antitumor activity of ZnNPs against Baby Hamster Kidney Fibroblast cells

The in vitro anticancer cytotoxic activity of (Zn-NPs) was investigated on baby hamster kidney fibroblast cell line (BHK-21) using sulforhodamine-B (SRB) assay. The cytotoxic activity was expressed as reduction of cell viability relative to control. The results were indicated in Fig. 2. The 50% growth inhibitory concentration (${\mathrm{IC}}_{50}$) value for ZnNPs was estimated from the available cytotoxicity. The ZnNPs recorded ${\mathrm{IC}}_{50}$ value 68.03 µg/ml against hamster kidney fibroblast cell line.

#### Biochemical Results

The effect of Zn-NPs on MDA level and antioxidant status (GSH content, GPx, and SOD enzyme activities) in the kidney tissue of different studied groups is reported in Table 1. Our results revealed a significant decrease in GSH content, GPx, and SOD activities while plasma MDA level was significantly increased in Fe-NTA-treated group upon their comparison with the normal control group. Whereas, Zn-NPs-treated rats (Fe-NTA + Zn-NPs) showed a significant increase in antioxidant status associated with a significant decrease in MDA content compared with Fe-NTA-model.

**Table 1:** Effect of Zn-NPs on the MDA level and antioxidant statue in the kidney tissue of different studied groups

Parameters	Groups			
	Control	Fe-NTA	Zn-NPs	Fe-NTA + ZnNPs
MDA (µmol/l)	158.00 ± 1.00^b^	217.65 ± 1.33^a^	170.64 ± 3.17^b^	173.33 ± 2.34^ab^
GSH (U/g)	107.00 ± 3.00^b^	80.6 ± 0.66^a^	97.0 ± 1.03^b^	94.3 ± 2.34^ab^
GPx (U/g)	0.5 ± 0.03^b^	0.33 ± 0.01^a^	0.48 ± 0.04^b^	0.45 ± 0.02 ^b^
SOD (U/g)	10.3 ± 0.81^b^	8.9 ± 0.13^a^	10.01 ± 0.53^b^	10.15 ± 0.42^b^

The mean difference is significant at the 0.05 level. *P* < 0.05

^a^Significant difference versus control group

^b^Significant difference versus Fe-NTA group

Results of plasma toxicity markers (Na^+^, K^+^, urea, and creatinine levels) as well as enzyme activities of ALT, LDH, and GGT are shown in Table 2. It was found that there is a significant increase of all previous parameters in Fe-NTA-treated group compared to the control group, whereas the results represented in the same table showed that plasma albumin and total protein concentrations in Fe-NTA group were significantly decreased with respect to control group. On contrary, Fe-NTA + Zn-NPs-treated rats showed a significant decrease in the levels of studied parameters compared to induced group. On the other hand, tumor-induced rat group treated with Zn-NPs showed a significant increase in both plasma albumin and total proteins concentrations upon its comparison with Fe-NTA model.

**Table 2:** Effect of Zn-NPs treatment on plasma toxicity markers of different studied groups

Parameters	Groups			
	Control	Fe-NTA	Zn-NPS	Fe-NTA + ZnNPs
Na (meq/l)	160.0. ± 0.57^b^	170.6 ± 0.66^a^	165.6 ± 0.33^ab^	165.8 ± 0.72^ab^
K (mmol/l)	5.4 ± 0.28^b^	7.4 ± 0.04^a^	5.7 ± 0.21^b^	5.6 ± 0.08^b^
Albumin (g/dl)	4.33 ± 0.06^b^	3.65 ± 0.02^a^	4.00 ± 0.10^ab^	4.06 ± 0.14^b^
Total protein (g/dl)	7.08 ± 0.11^b^	6.05 ± 0.07^a^	7.04 ± 0.11^b^	6.90 ± 0.09^b^
ALT (U/ml)	43.32 ± 5.23^b^	66.64 ± 3.32^a^	49.00 ± 2.30^b^	50.32 ± 2.90^b^
LDH (U/ml)	849.6 ± 5.78^b^	881.50 ± 4.44^a^	858.00 ± 4.61^b^	860.00 ± 2.87^b^
GGT (U/g)	12.66 ± 0.88^b^	18.83 ± 0.16^a^	13.33 ± 0.33^b^	14.65 ± 0.88^b^
Urea (mmol/l)	29.0 ± 0.57^b^	56.6 ± 2.40^a^	33.0 ± 1.15^b^	34.6 ± 1.76^ab^
Creatinine (mg/dl)	0.54 ± 0.02 ^b^	1.22 ± 0.15^a^	0.76 ± 0.03^b^	0.78 ± 0.07^b^

The mean difference is significant at the 0.05 level. *P* < 0.05

^a^Significant difference versus control group

^b^Significant difference versus Fe-NTA group

**Fig. 3:**
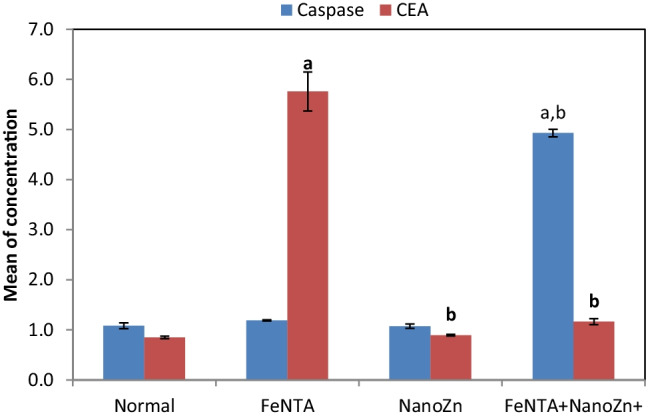
Effect of Zn-NPs treatment on the levels of caspase-3 (U/ml) and CEA levels (ng/ml). Results are given as mean ± SE. Means are significantly different at *p* < 0.05 (a) Significance compared to control group. (b) Significance compared to Fe-NTA group

Caspase-3 level in the kidney tissue homogenate recorded a significant increase in the Fe-NTA + Zn-NPs group upon their comparison with the normal control and Fe-NTA groups. Plasma CEA was significantly increased in Fe-NTA-treated rats compared to control group; however, Zn-NPs treatment (Fe-NTA + Zn-NPs group) resulted in a significant decrease in CEA level compared to Fe-NTA model (Fig. 3).

#### DNA Fragmentation

**Fig. 4:**
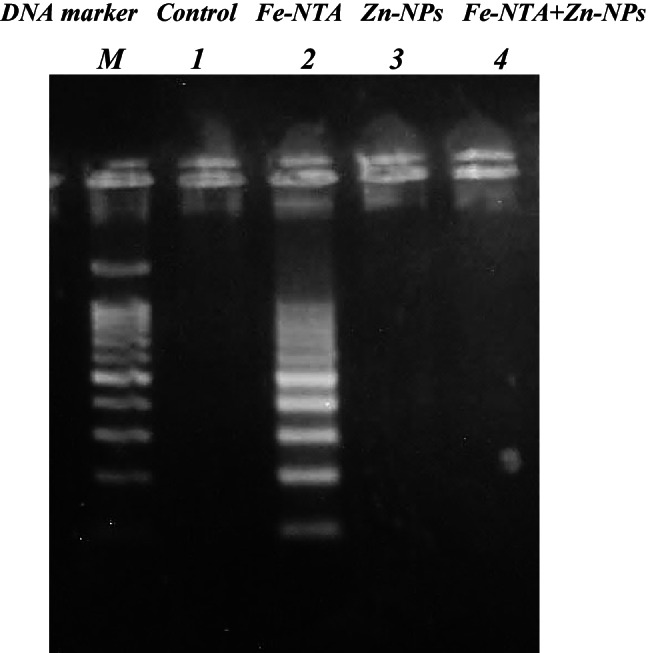
DNA fragmentation pattern of different studied groups on agarose gel electrophoresis. Necrotic strand breaks/streaking DNA was observed in Fe-NTA group (lane 2), but not in other groups (lanes 1, 3, 4)

Analysis of agarose gel electrophoresis revealed that DNA was completely intact in kidney samples of both normal control and Zn-NPs groups. However, induction of renal carcinoma resulted in elevation in the degree of DNA fragmentation in kidney samples of Fe-NTA group; treatment with Zn-NPs succeeded in repairing the fragmented DNA compared to renal induction samples (Fig. 4).

#### Histopathological Examination

**Fig. 5:**
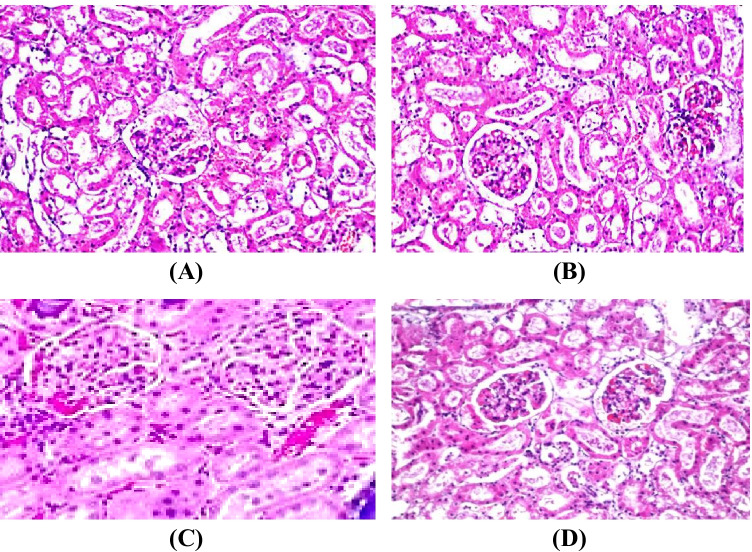
Hematoxylin and eosin–staining histological examinations of rat kidney sections representative of the antitumor efficacy of Zn-NPs in renal cancer induced by Fe-NTA in Wistar rats from different groups (40 × magnification). **A** Control group: there was no histopathological alteration, and the normal histological structure of the glomeruli and tubules at the cortex were recorded. **B** Zn-NPs group: vacuolization was observed in the lining endothelium of the congested glomerular tufts. **C** Fe-NTA- intoxicated group: there was congestion in their glomerular tufts associated with degeneration and dysplasia and disfiguration in the lining epithelium of the tubules. **D** Fe-NTA + Zn-NPs group: congestion was detected in the glomeruli associated with mild dysplasia in the lining tubular epithelium

Histopathological examination of kidney sections derived from normal control rats (Fig. 5A) and Zn-NPs-treated rats (Fig. 5B) showed normal histological structure of the glomeruli and tubules at the cortex. However, the examined kidney sections from Fe-NTA rats revealed congestion in their glomerular tufts associated with degeneration and dysplasia and disfiguration in the lining epithelium of the tubules (Fig. 5C). The examined kidney sections from Zn-NPs-treated rats after intoxication with Fe-NTA revealed congestion were detected in the glomeruli associated with mild dysplasia in the lining tubular epithelium (Fig. 5D).

## Discussion

The present study was designed to assess the antitumor activity of green biosynthesized Zn-NPs as therapeutic agent against renal cancer induced by Fe-NTA in rats. Results from our biochemical analysis reveal that the levels of CEA, MDA, Na + , K + , urea, creatinine, ALT, LDH, and GGT were significantly decreased, while the levels of SOD, GSH, GPx, and caspase-3 were significantly increased in Fe-NTA + Zn-NPs-treated rats when compared to Fe-NTA-treated rats. These results show the protective antitumor activity of Zn-NPs which emphasized by histopathological examination and DNA fragmentation. In our study, Zn-NPs were biosynthesized from the edible white mushroom *Agaricus bisporus*; this process is known as green synthesis of nanomaterials which proved to have many benefits for the environmental ecosystem and also very useful in controlling the desired size and shape for nanomaterials [24–26]. This study is considered from a few studies which prepared Zn-NPs, as most studies prepared Zinc oxide nanoparticles (ZnO-NPs) which proved to have cytotoxic effects [13–16]. In this study, the green biosynthesized Zn-NPs were very safe and had no toxic effects, as results of LD50 revealed that no toxicity symptoms and no mortality were recorded, even Zn-NPs were used at a dose of 1000 µg/kg b.w.

The obtained Zn-NPs were characterized by different analytical methods. Transmission electron microscopy (TEM) photos of Zn-NPs revealed that shapes were found to be spherical and size ranging from 12 to 17 nm. Zetasizer particle size analysis using dynamic light scattering (DLS) for Zn-NPs revealed that Zn-NPs ranged from 50–100 nm. This result is in agreement with result obtained by others [27] who reported that TEM of biogenic Zn-NPs synthesized by Lavandula Vera leaf extract were found to be of spherical shape with the size range of 30–80 nm, and the most frequent nanoparticles (NPs) were in the range of 50–60 nm.

Generally, formation of the nanoparticles assisted by different extracts led to addition of some functional groups on the surface of nanoparticles. Thus, Fourier transform infrared spectroscopy (FTIR) measurements were used to recognize such functional groups located on the surface of biogenic Zn-NPs [28]. In the present work, FTIR analysis of Zn-NPs and biological material used in the synthesis (mushroom extract) showed shifting in the signal wave number cm^−1^ of hydroxyl group when compared that of biological extract with Zn-NPs indicates the involvement of –OH in Zn-NPs formation and stabilization as it is used in the zinc ion reduction and capping forming Zn-NPs and the presence of functional groups as in the mushroom FTIR. These results are in agreement with result obtained by previous study [27] that identified strong FTIR peaks functional groups in the plant extract as found in Zn-NPs FTIR analysis consistent with the results obtained in FTIR of the present study.

In this study, the renal cancer in rats was induced by using Fe-NTA twice weekly at a dose of 9 mg/kg b.w. for 9 months (the end of experiment). Fe-NTA is a potent nephrotoxic agent, causing renal adenocarcinoma in experimental rats when administrated for several times [29]. The mechanism by which Fe-NTA causes its effects seems mainly due to its ability to deplete the antioxidant battery and induce ROS generation by iron-catalyzed Fenton reaction, which in turn causing lipid peroxidation, and DNA damage [30, 31]. This is consistent with our results as the level of MDA was significantly increased while the level of GSH and the activities of SOD and GPx were significantly decreased in Fe-NTA-treated rats when compared to controls. Also, results obtained from DNA fragmentation assay revealed the elevation in the degree of DNA fragmentation in kidney samples of Fe-NTA group. When rats were received Fe-NTA then treated with Zn-NPs, it was well-noticed that levels of MDA and DNA fragmentation were significantly decreased while the level of GSH, and the activities of SOD and GPx were significantly increased in Fe-NTA + Zn-NPs-treated rats when compared to Fe-NTA-treated rats, confirming the protective effect of Zn-NPs against the oxidative stress induced by Fe-NTA. In agreement to our findings, several studies reported that Fe-NTA enhance lipid peroxidation in kidney tissue by increasing thiobarbituric acid reactive substances such as MDA which is cytotoxic, damage the DNA leading to 8-hydroxy-2′-deoxyguanosine (8-OHdG) formation of which is mutagenic and carcinogenic leading to numerous functional changes in cells [31–33].

Renal cancer lacks specific predictive biomarkers, and only some symptoms, such as hematuria, might aid in discovering the presence of cancer [34]. Therefore, the authors choose two nonspecific tumor markers: CEA and LDH, to monitor the antitumor effect of Zn-NPs. Results of the current study showed that serum levels of both CEA and LDH were significantly decreased in Fe-NTA + Zn-NPs-treated rats when compared to Fe-NTA-treated rats, demonstrating the antitumor effect of Zn-NPs. Serum levels of CEA are known to be elevated in different types of cancer including renal cancer [35, 36]. Cancerous cells are reported to have upregulated LDH, and elevated LDH levels are associated with poor outcomes in cancer patients [37]. Also, previous studies suggest the role of LDH in tumor progression [38, 39].

Caspases are a family of 15 protease enzymes playing essential roles in programmed cell death i.e., apoptosis [40]. Caspase-3 is the main executioner of apoptosis, it is activated by other caspases (-8, -9, or -10). Once activated, it is stimulating the proteolytic cleavage of other proteins which is related to fragmentation of DNA, nuclear collapse, and condensation and margination of chromatin [41, 42]. Many anticancer therapeutic agents are able to cause tumor cell death by activating caspase-3; therefore, caspase-3 activation is used as a surrogate marker for the efficacy of cancer treatment [43]. Results of the present study show that the levels of caspase-3 in the kidney tissue homogenate were significantly increased in the Fe-NTA + Zn-NPs group upon their comparison with the normal control and Fe-NTA groups, confirming the antitumor efficacy of Zn-NPs in renal cancer treatment.

Concomitant with the improvement of the biochemical markers and DNA fragmentation, the histopathological examination of kidney sections from Zn-NPs-treated rats after intoxication with Fe-NTA revealed an improvement by noticeable amelioration in the cellular architecture of kidney tissue with mild dysplasia in the lining tubular epithelium.

## Conclusion

From the aforementioned results, it can be concluded that Zn-NPs ameliorate the oxidative stress induced by Fe-NTA, which is manifested by a decrease in lipid peroxidation, increase of enzymatic and non-enzymatic antioxidant molecules as well as it possesses antitumor promoting activity against Fe-NTA-induced toxicity and carcinogenesis through reduction of tumor cell infiltration and enhancing tumor cell apoptosis which is confirmed histopathologically and biochemically by decreased CEA, increased caspase-3, and decreased level of DNA fragmentation. Therefore, Zn-NPs are considered a good therapeutic agent against renal cancer induced by Fe-NTA in rats.

## Data Availability

All data generated or analyzed during this study are included in this published article.
